# Accelerated flexible protein-ligand docking using Hamiltonian replica exchange with a repulsive biasing potential

**DOI:** 10.1371/journal.pone.0172072

**Published:** 2017-02-16

**Authors:** Katja Ostermeir, Martin Zacharias

**Affiliations:** Physik-Department T38, Technische Universität München, Garching, Germany; University of Michigan, UNITED STATES

## Abstract

A molecular dynamics replica exchange based method has been developed that allows rapid identification of putative ligand binding sites on the surface of biomolecules. The approach employs a set of ambiguity restraints in replica simulations between receptor and ligand that allow close contacts in the reference replica but promotes transient dissociation in higher replicas. This avoids long-lived trapping of the ligand or partner proteins at nonspecific, sticky, sites on the receptor molecule and results in accelerated exploration of the possible binding regions. In contrast to common docking methods that require knowledge of the binding site, exclude solvent and often keep parts of receptor and ligand rigid the approach allows for full flexibility of binding partners. Application to peptide-protein, protein-protein and a drug-receptor system indicate rapid sampling of near-native binding regions even in case of starting far away from the native binding site outperforming continuous MD simulations. An application on a DNA minor groove binding ligand in complex with DNA demonstrates that it can also be used in explicit solvent simulations.

## Introduction

Protein-ligand complex formation triggers the majority of biological processes in cells and mediates the effect of drug molecules. The identification of possible ligand binding sites on biomolecules is important for the prediction of ligand-receptor binding geometry and for specific ligand design. Computationally effective widely applied molecular docking methods are based on a systematic docking search largely neglecting conformational flexibility or including flexibility of binding partners only approximately [1,2,3,4,5]. During a systematic search stage empirical scoring functions are employed that allow rapid scoring of generated complexes based on surface complementarity and pairwise interaction potentials [4,5]. However, complex formation can directly induce local conformational changes in a receptor protein such as side chain or protein loop rearrangements but can also cause global changes that correspond to adjustments of secondary structural elements or whole domains [2,6]. Typically, in a second step docking solutions are refined using methods that allow for conformational changes of the partner molecules (e.g. molecular dynamics simulations) and possible rescoring of selected solutions on a more sophisticated level compared to the initial systematic screen [3,7] and including full ligand and receptor flexibility [2,8,9].

The success of such a two step scheme with an initial rigid docking followed by flexible refinement depends heavily on the result of the initial systematic search. Ideally, complex geometries should be generated with a method that allows for full conformational flexibility of both binding partners on an atomistic level and includes explicit solvent during the entire search for binding sites on the biomolecular surface. In principle, Molecular Dynamics (MD) simulations are ideally suited, since atomic level flexibility and inclusion of explicit water molecules are possible[10].

Indeed such simulations have been performed on several model systems [10,11,12] to study the complete association process in atomic detail either using hundreds of simulations each in the range of microseconds [13,14] or ultra-long simulations reaching the millisecond regime [15,16]. It is possible to predict the binding process of ligands to their target molecules as well as kinetics and energy barriers [15]. Characteristic for such simulations is the transient binding of ligands to “sticky” suboptimal (non-specific) sites which can be occupied during a large fractions of simulation time [14,16]. Hence, in order to rapidly and systematically search for binding sites of protein-protein or protein-drug complexes, such approaches are still computationally too demanding. It is possible to couple MD-simulations of ligand-receptor binding with metadynamics to predict binding geometries and to also obtain an estimate of the free energy of binding [17]. However, the ligand binding site on the protein surface needs to be known approximately and a biasing potential to compensate for the ligand-receptor interaction is built up gradually in a time-consuming process. Related to this approach it is possible to use reconnaissance metadynamics to search for ligand binding sites where again a biasing potential is gradually introduced to destabilize already visited binding regions on the surface of a receptor molecules[18].

Sampling can also be improved in replica-exchange Molecular Dynamics (REMD) simulations employing a set of parallel MD simulations running at different temperatures [19,20,21]. Conformations, sampled in neighboring replicas are allowed to exchange after predefined time intervals and can also improve the sampling in the reference replica. However, the efficiency of the standard temperature (T)-REMD depends significantly on the size of the system, in addition, it does not enhance sampling in case of entropic barriers. In Hamiltonian (H)-REMD simulations parts of the force field are modified along the replicas [22,23,24]. Exchanges between simulations controlled by different force fields are accepted or rejected according to a Metropolis criterion and allow conformations to travel through different replica conditions to finally accelerate conformational sampling in the reference replica under the control of the original force field. H-REMD can outperform T-REMD because of focusing on a relevant degree of freedom of a system instead of affecting all atoms of the system in T-REMD [22].

REMD methods have been successfully applied for structural refinement of proteins in complex with small organic ligands [20,24]. In a combined experimental and Replica Monte Carlo study, Kim et al. characterized transient ligand-receptor interactions in the encounter complexes, by promoting rigid body motion and interacting side chain flexibility on a coarse grained level [20]. In other H-REMD based docking studies Coulomb and Lennard Jones interactions between ligand are scaled along the replica ladder in implicit solvent [23] or in explicit solvent [24] for predicting the correct binding geometry. However, the weakening of the steric interactions between ligand and receptor in such approach may result in sampling of many irrelevant states (with atom overlaps) in the higher replicas. These states do not affect the canonical sampling with respect to the original force field in the reference replica but may decrease search efficiency. It was found in a previous study of our lab that scaling/softening the non-bonded interactions along the replicas in a H-REMD is very helpful to overcome barriers in the vicinity of a ligand binding site but can still result in kinetically trapped states if started from an initial placement far from the native binding region (24).

In this work we present a H-REMD-based docking protocol to accurately and rapidly predict both native binding sites and binding poses of protein-protein and other protein-ligand complexes. It is intended to simultaneously screen large surface areas of putative binding regions of interaction partners and to locally refine complex geometries in the correct binding site in explicit or implicit solvent. One major problem of using MD-simulations for identifying possible ligand-receptor binding sites is the possibility of becoming trapped in a locally stable binding mode requiring long associated simulation times to escape from such non-native binding regions. In the present approach we try to circumvent this problem by employing a biasing potential that penalizes the smallest contact distance between two partner molecules by means of an ambiguity restraint. This type of restraint assigns weights to a set of possible contacts between surface atoms of the partners with the highest weight assigned to the shortest distance. By modifying this ambiguity restraint along the replicas a gradual increase in the average distance between the ligand and the receptor is achieved. While moving in close proximity to the protein surface in the reference replica, ligands can rotate and translate freely at slightly larger distances around the receptor surface in the higher replicas. In spirit this is similar to the reconnaissance metadynamics approach [18], however, in the present approach a biasing potential (at different levels) is already present from the beginning on of the REMD simulation that penalizes binding and a time consuming build up procedure that may also lead to a complicated biasing potential with many stored terms is avoided. The biasing potential in the replicas allows the ligands to escape from suboptimal binding sites to effectively search the protein surface. The approach was tested on several protein ligand complexes with ligands of different sizes and types and allowed the identification of the native complex in reasonable computer time (few hours on a parallel computer) even when starting far from the native binding region. Thus, the approach may offer a route for exhaustive search for binding sites including flexibility of binding partners as well as solvent either by an implicit or explicit description.

## Materials and methods

### Implicit solvent molecular dynamics simulations and test sytems

All simulations were performed by means of the *pmemd* module of the Amber14 package [25] using the ff14SB force field [26]. Simulations employing an implicit Generalized Born (GB) solvation model were performed with the OBC (Onufriev, Bashford, and Case) GB model [27] (igb = 5 in Amber input file) and a 12 Å Born radius cutoff combined with a cutoff for electrostatic calculations of 20 Å. Hydrogen mass re-partioning combined with the shake algorithm for solute bonds involving non-hydrogen atoms allowed for a 4 fs timestep [28]. The systems were run at constant temperature (280–300 K) using a Langevin thermostat with collision frequency of 0.1 ps ^−1^ (reduced viscosity) [29]. As the most simple test system (I) a 3-helix homedomain structure was used (pdb2sy9: 3^rd^ homeodomain from human homeoz protein). The sequence was truncated between helix-2 and helix-3 resulting in an artificial single helix ligand (third helix: residues 47–58 of pdb2sy9: TQQVLDWFDSRL) and residues 16–44 (helix-1 and helix2: PDIQPLERYWAAHQQLRETDIPQLSQASR) forming an artificial small receptor protein. In the native helix arrangement the third helix forms a complex with the two other helices with a well packed (native) interface. For this system a single most difficult start structure at maximum distance on the receptor surface was generated with the ligand helix placed opposite to the native binding site. The second system (II) consisted of a homodimeric protein-protein complex (chains A and B of pdb2oo9) representing one of the smallest protein-protein complexes (with 44 and 46 residues for the two partners, respectively, resolved in the X-ray structure). For the docking simulations the two partners were separated and placed at five random positions and orientations of one partner (ligand) close to the surface of the other protein partner (treated as receptor protein). The FK1 domain of the protein FKBP51 (FK506 binding protein 51) in complex with the FK506 ligand (126 atoms) served as a third test system [30]. The parameters of the FK506 ligand were obtained by means of the antechamber module of Amber using the semi-empirical bcc option for calculating partial charges. Similar to the system II starting structures were generated by random rotation and translation of the FK506 ligand relative to the FKBP51 protein. Each starting geometry was first energy minimized including positional restraints on heavy atoms (5000 steps steepest descent) and then were gradually heated from 100 to 300K in 3 consecutive 0.4 ns simulations. In five additional 0.4 ns simulations positional restraints were gradually removed, resulting in equilibrated start structures for the RE-DOCK simulations.

### Explicit solvent molecular dynamics simulations on DNA in complex with a minor groove ligand

The binding and movement of a berenil-analogue (2,5-bis-(4-guanylphenyl)furan) minor groove binder (BGF) in complex with a 18 base pair B-DNA (sequence: 5’-GCGCAATTGCGTCAGCGC) was studied after placing the ligand in the minor groove at the center of the helix. The DNA helix was generated using the NAB module of the Amber14 package. The parameters of the minor groove ligand were obtained by means of the antechamber module (see above). The ligand was placed at the center of the helix after superimposing an experimental BGF-complex with DNA (pdb227d) onto the central part of the 18 bp dsDNA. After superposition only the ligand and 18 bp dsDNA were retained and energy-minimized (5000 SD steps). Note, that the central segment of the DNA is GC rich which does not correspond to the preferred AT-rich binding region of the minor groove ligand. The structure was solvated in a rectangular box with explicit TIP3P water molecules (minimum distance of 10 Å between box boarder and any atom of the solute) and neutralized with sodium ions by means of the Amber14 leap module and using the parmbsc0 force field for DNA [31]. Long range electrostatic interactions were calculated with the Particle Mesh Ewald (PME) method [32] and a real space cutoff radius of 9Å. Hydrogen mass repartitioning allowed for a time step of 4 fs. During 0.5 ns equilibration in steps of 100 K the system was heated up to 300 K while protein heavy atoms were harmonically restrained (25 kcal mol^-1^ Å^-2^) to positions in the starting structure. Subsequently, positional restraints were gradually removed during another 0.5 ns at 300 K and constant pressure (1 bar). During production runs to keep the long dsDNA approximately aligned along the long axis of the box the heavy atoms of the DNA were very weakly restraint to the positions in the starting (B-DNA) structure using a force constant of 0.005 kcal mol^-1^ Å^-2^. This still allows large conformational freedom of all DNA atoms but avoids large rotations of the DNA with respected to the long axis of the box. Simulation results were analyzed by means of the cpptraj module of Amber14.

### Construction of the biasing potential for H-REMD simulations

The purpose of the biasing potential used in the present simulation is to penalize trapping of the simulations in possible local minima on the surface of the partner molecules at the many possible “sticky” sites on protein surfaces. This is achieved by an ambiguity distance restraint [33] which involves groups of atoms of one partner (N_r_ atoms in one partner) and the other partner (with N_l_ atoms). The mean distance between groups of atoms calculated from an ambiguity restraint is typically obtained as:

$$d_{ave-6}={<d_{rl}^{-6}>}^{-1/6}={\left[\frac{1}{N_{r}N_{l}}\sum_{r=1}^{N_{r}}\sum_{l=1}^{N_{l}}\frac{1}{d_{rl}^{6}}\right]}^{-1/6}$$

It involves the calculation of all intermolecular distances between the groups of atoms in one and the other partner (see group definition below). Instead of calculating a simple mean distance the mean of 1/d_rl_^-6^ is calculated giving a short distance a much higher weight than large distances. In fact, the calculated weighted mean distance d_ave-6_ is dominated by the shortest distances d_rl_ between members of the groups (N_r_ and N_l_). Note, that the shortest distance will constantly change during the simulation. In order to further increase the dominance of the shortest distances we calculated the ambiguity distance d_ave-12_ in the pmemd code using:

$$d_{ave-12}={<d_{rl}^{-12}>}^{-1/12}={\left[\frac{1}{N_{r}N_{l}}\sum_{r=1}^{N_{r}}\sum_{l=1}^{N_{l}}\frac{1}{d_{rl}^{12}}\right]}^{-1/12}$$

The necessary code modifications in the Amber pmemd program and an example are provided in Supporting Information S1 File. For the definition of the atom groups on both partners (in the starting conformation) only surface (heavy) atoms were considered as identified by a rolling sphere algorithm [34] to include surface atoms with a minimum accessible surface of 10 Å^2^ (probe radius 1.5 Å). In a second step we restricted this selection to atom pairs of the receptor and the ligand that are less than 10 Å apart from each other to finally calculate d_ave-12_. This second selection is updated every simulation step. If the distance between each atom pair of the ligand and receptor exceeds 10 Å, a simple harmonic distance restraint was put on the atom pair with closest distance. Thus, the two binding partners could not dissociate. A biasing potential H_i_ was added to each replica in the H-REMD simulations that allowed or penalized different regimes of the d_ave-12_ between the two partner molecules with the general form:

$$H_{i}(d_{ave-12})=\begin{array}{c} k_{f}{\left(d_{ave-12}-d_{ave-12,low}\right)}^{2}\;if\;d_{ave-12}\leq d_{ave-12,low}\\ k_{f}{\left(d_{ave-12}-d_{ave-12,up}\right)}^{2}\;if\;d_{ave-12}\geq d_{ave-12,up}\\ 0\;if\;d_{ave-12,low}<d_{ave-12}<d_{ave-12,up} \end{array}$$

where k_f_ = 2.5–5 kcal mol^-1^ Å^-2^

**Fig 1:**
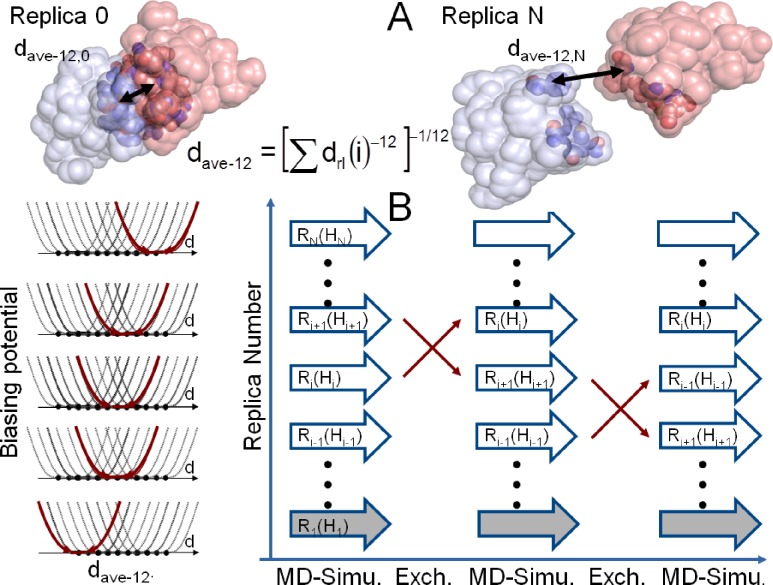
Illustration of the RP-REMD-Dock approach. (A) Based on the accessible surface of the two isolated partner molecules two surface groups of atoms are defined. The 1/d^-12^ weighted distance is mainly determined by the closest contacts between the two groups and is used to define biasing penalty potentials for each replica (illustrated in B). The harmonic potentials allow for each replica a range of closest distances between the two partners such that the lower replicas allow close contacts without penalty but the higher replicas penalize such contacts by a quadratic function and push the partners on average slightly away from each other. The actual quadratic biasing potential in each replica run is illustrated in red (bold line), the range of potentials is indicated a thin lines. Since the allowed distance intervals overlap significantly between neighboring replicas a high acceptance rate for Hamiltonian replica exchanges leads to quick exploration of the biomolecular surfaces (replica scheme in the right panel).

During the BP-REMD any arrangement between partners is possible as long as the d_ave-12_ is within the allowed interval (given by d_ave-12,low_ and d_ave-12,up_ for each replica). As explained above the calculated d_ave-12_ is dominated by short range distances: If, for example, one or more short distances are established between the partners such that the calculated d_ave-12_ is smaller than the preset lower limit in the replica (d_ave-12,low_) the penalty term becomes active (controlled by the force constant given above) resulting in a force to increase the distance between partners (due to the construction of the weighted average distance the largest force contribution will act on the shortest contact distance between atoms of the partner molecules). The result is that the partners are repelled in the replica run until the weighted distance is again within the allowed interval. For the reference replica the lower bound is such that any contact between the two partner molecules is possible without penalty. The lower and upper bounds for d_ave-12_ increase with the replica number such that still overlap of sampled states between neighboring replicas is possible but a close contact between partner molecules is significantly penalized in the higher replicas (Fig 1). To prevent the binding partners from dissociating in the higher replicas an additional potential contribution keeps the 2 closest atoms of both binding partners at a minimal distance of 10 Å (only if d _ave-12_ >10 Å). For the initial test case with 6 replicas we used the following intervals for d_ave-12,low_ / d_ave-12,up_: replica 1: 4.0/6.0 Å; replica 2: 5.15/7.15 Å; replica 3: 5.65/7.65 Å; replica 4: 6.05/7.15 Å; replica 5: 6.45/7.65 Å; replica 6: 6.75/8.00 Å. For the cases with 10 replicas, d_ave-12,low_ / d_ave-12,up_: replica 1: 4.0/6.0 Å; replica 2: 4.5/6.5 Å; replica 3: 5.0/7.0 Å; replica 4: 5.5/7.5 Å; replica 5: 6.0/8.0 Å; replica 6: 6.5/8.5 Å; replica 7: 7.0/9.0 Å; replica 8: 7.5/9.5 Å; replica 9: 8.0/10.0 Å; replica 10: 8.5/10.5 Å. In case of the explicit solvent simulations with 12 replicas a set of d_ave-12,low_ / d_ave-12,up_ intervals: 1: 4.0/6.0 Å; 2: 4.50/7.15 Å; 3: 5.0/7.65 Å; 4: 5.3/7.5 Å; 5: 5.7/8.0 Å; 6: 6.0/8.5 Å; 7: 6.3/9.0 Å; 8: 6.7/9.0 Å; 9: 7.0/9.0 Å; 10: 7.3/9.0 Å; 11: 7.7/9.0 Å; 12: 8.0/9.0 Å was used. Test simulations indicated that these intervals resulted in exchange acceptance rates for the H-REMD of ~50% which allowed also a high turn around in the whole set of H-REMD simulations.

### Protein flexibility

The use of an implicit solvent model during MD simulations may not always favor the native structure of the binding partners and can even cause unfolding of proteins. In order to prevent unfolding of the binding partners during implicit solvent MD simulations and to interfere with our binding simulations we included positional restraints of the backbone C_α_ atoms for one partner (receptor, force constant 0.5 kcal mol^-1^ Å^-2^). For the protein-protein interaction case (pdb2oo9) the distance of all C_α_ atom pairs was restraint to the corresponding values in the crystal structure (force constant 2.5 kcal mol^-1^ Å^-2^). This allows complete rotational and translational freedom of the ligand with respect to the receptor and also full side chain flexibility but only limited backbone flexibility. In case of the peptidomimetic fkbp ligand only the receptor protein backbone was restraint to the X-ray reference structure whereas the ligand was fully flexible.

## Results and discussion

**Fig 2:**
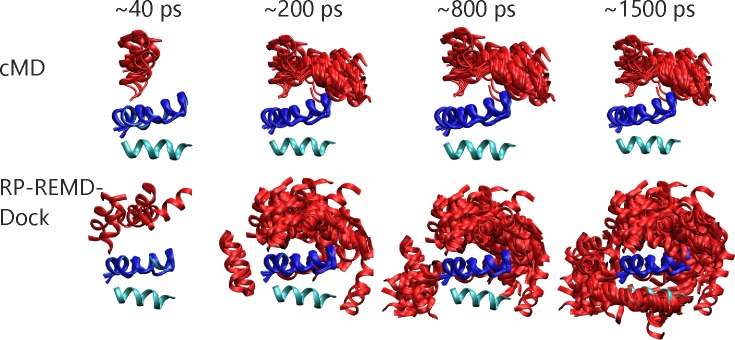
The artificial “homeodomain” test case consists of a single ligand helix (light blue cartoon) that corresponds to the third helix of 3^rd^homeodomain from human homeoz protein (pdb2sy9) whereas the receptor was formed by the remaining helix 1 and 2 segments (dark blue cartoon). The loop segment between helix 2 and helix 3 in the original structure was eliminated to yield a complex of two peptide molecules. For the MD simulations the ligand helix was initially placed on the opposite site of the native placement and snapshots of sampled states after 20, 200, 800 and 1500 ps are indicated (red cartoon). The upper panels indicate snapshots obtained during regular MD simulation. Lower panels are snapshots found in the RP-REMD-Dock reference replica. At the final stage sampling (last panel on the right) several of the sampled configurations overlap with the native placement of the helix 3 ligand as found in the original homeodomain structure.

The application of Molecular Dynamics (MD) simulations to investigate the binding process between biomolecules offers the possibility to include various levels of conformational flexibility and the currently most accurate inclusion of the solvation effects by inclusion of explicit solvent molecules. However, the interaction surface of proteins and other biomolecules contains a variety of polar and nonpolar groups that can transiently interact with chemical groups of other biomolecules. Unfortunately, during MD simulations of biomolecular encounter processes such “sticky” sites can result in many locally trapped binding states that are separated by barriers with long associated life times. If these life times exceed or are in the same order as the simulation time there is little chance to reach the native binding mode. For a simple test system consisting of a helical peptide ligand in complex with an artificial 2-helix bundle receptor this is illustrated in Fig 2 (see also first paragraph of the Methods section). During standard MD simulations each partner was restraint to a conformation close to the bound form by imposing distance restraints between heavy atoms for the ligand and positional restraints with respect to a reference structure for the receptor (see Methods). For this system such restraints were necessary to prevent unfolding of the partners and to keep the native binding mode as the most favorable configuration (not the case without the distance restraints).

When starting from an arrangement with the ligand placed on the side of the receptor opposite to the native binding site cMD simulations became trapped at positions far from the native binding mode (Fig 2). In contrast in a RP-REMD-Dock simulation with six replicas one can see a much broader exploration of possible binding states in the reference replica (that does not penalize contacts) compared to cMD in the same simulation time (Fig 2). Since in the higher replicas the distance of the closest atoms of the partners is penalized to avoid any close contacts rapid motion (translation and rotation) of the binding partners relative to each other is possible. Due to the high exchange rate between neighboring replicas in the RP-REMD-Dock any favorable configuration which allows close contacts can exchange to populate the reference replica.

**Fig 3:**
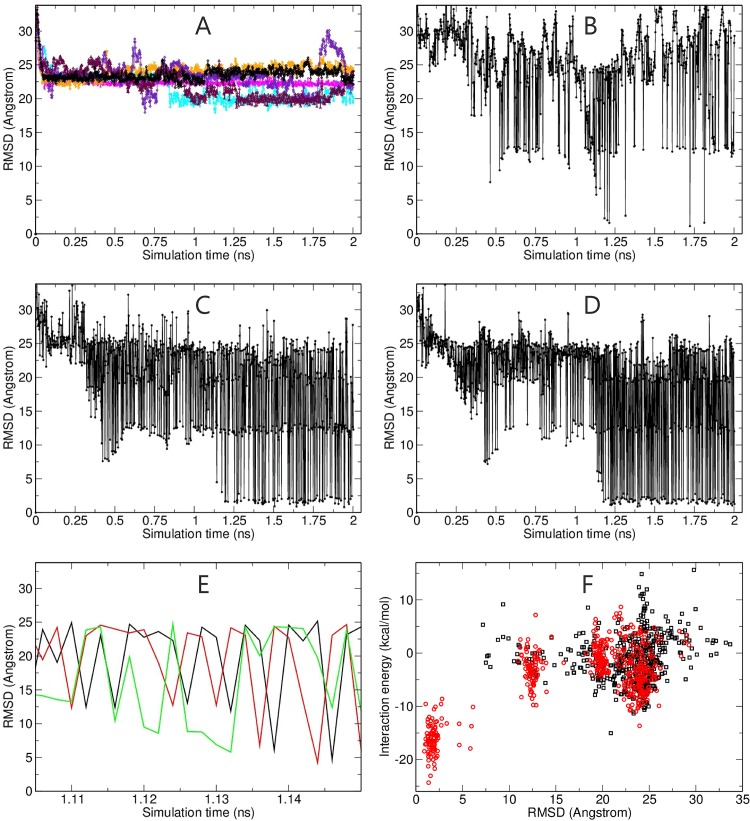
(A) Root mean square deviation of the ligand (RMSD_lig_) helix (backbone atoms, after superposition of the receptor part onto the reference) from the native position in the homeodoman arrangement (pdb2sy9) during six MD simulations (each 2 ns, color coded lines) starting from the a placement on a side of the receptor opposite to the native binding position (see Fig 2). The RMSD_lig_ in case of the RP-REMD-Dock simulations in replica 6, 3 and in the reference replica (1) is indicated in B-D, respectively. (E) Enlarged view of the sampled states in the time regime around 1.1–1.15 ns for the RMSD_lig_ of replica 6 (green line), replica 3 (red line) and replica 1 (black line, reference replica) during the REMD simulation. (F) Calculated non-bonded interaction energy of the sampled complexes vs RMSD_lig_ of the complexes sampled in the reference replica. The black squares indicate the sampling during the first half of the BP-REMD whereas the red circles are the states sampled in the second final stage of the BP-REMD. Note, that the interaction energy was calculated from a re-evaluation of the sampled trajectory using an infinite cutoff for the electrostatics and the GB-model.

Eventually configurations close to the bound native binding mode are sampled and form the dominant sampled state in the reference replica after around 2 ns simulation time (Fig 3). The population of the native binding mode is also higher in the reference replica compared to the higher replicas (Fig 3D). However, Fig 3E also demonstrates that the higher replicas help to promote transitions to the native binding arrangement. The close-up view on the time interval 1.1–1.25 ns shows that a binding mode already close to the native binding arrangement with an RMSD_Lig_ ~5–6 Å is first sampled in the highest replica (green line in Fig 3E) before it first occurs in an “intermediate” replica and then exchange into the lowest reference replica (black line in Fig 3E). We also explored if the ligand-receptor interaction energy of sampled states could be used to identify near-native complex geometries. Indeed, the interaction energy of the near-native binding geometries sampled in the reference replica in the second half of the RP-REMD simulation is significantly more favorable than in case of non-native solutions with large RMSD_lig_ (Fig 3F).

**Fig 4:**
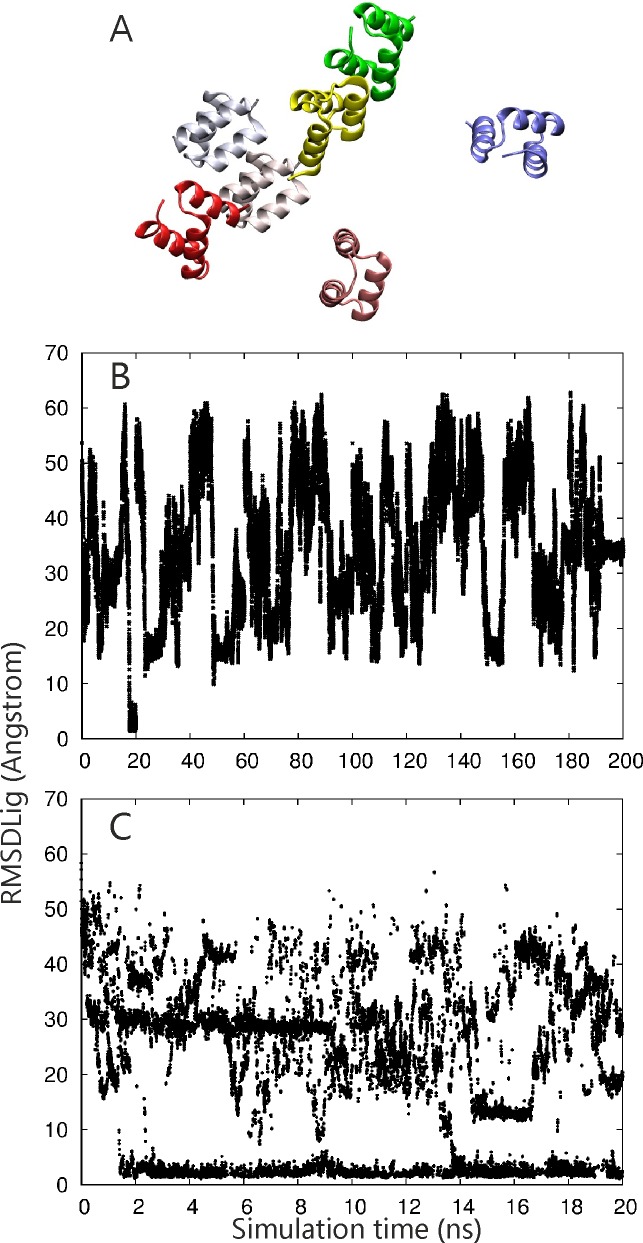
(A) Bound reference structure of the protein homodimer test case (receptor, chain A of pdb2oo9, as blue and ligand protein, chain B of pdb2oo9, as pink cartoon) and random starting placements of the ligand protein around the receptor (different colors of cartoons). (B) Sampled RMSD_lig_ during 10 MD simulations (same number as replicas used in the RP-REMD-Dock simulation) starting from the second start placement of the ligand protein. Each docking MD run was performed for 20 ns and results were concatenated for clarity to form a trajectory of 10x20ns = 200 ns resulting in a single overview plot (B). The sampled The RMSD_lig_ of the reference replica during RP-REMD-Dock simulations starting from the same initial placement as used in the MD simulations is illustrated in (C).

**Fig 5:**
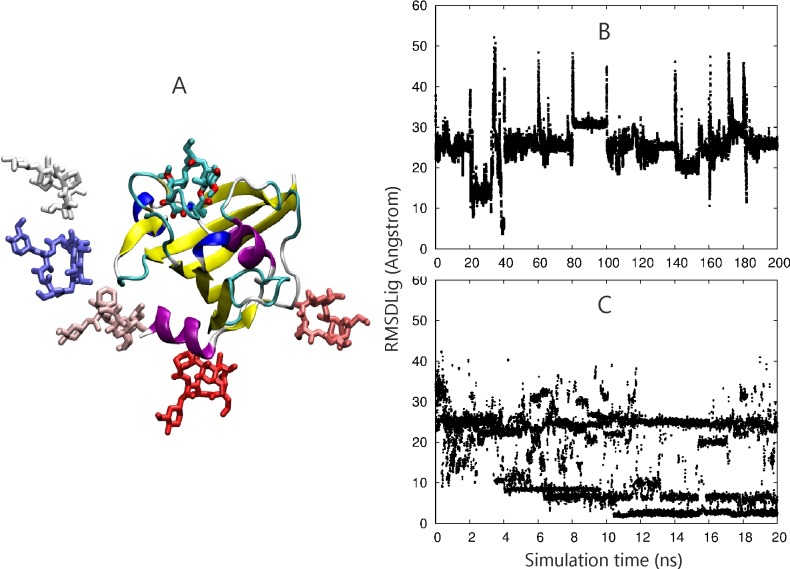
(A) Complex of FK506-ligand (atom color coded sticks) and the FKBP51 binding protein (color coded cartoon) and the initial ligand placements for the docking simulations are indicated (stick models in different colors). (B) RMSD_lig_ of FK506 (all heavy atoms with respect to the bound reference position after superposition of the sampled complex onto the reference receptor structure) for 10 independent MD simulations (each 20 ns concatenated into one long 10x20 ns trajectory). (C) same as in (B) but for the sampling in the reference replica of the RP-REMD-Dock simulations.

In a second more challenging application we performed docking MD simulations on a small protein-protein complex. The complex refers to a homodimer (chain A an B of pdb 2oo9, consisting of only 44–46 amino acids) that interact in a symmetric fashion. Similar to the first case an implicit solvent GB model was employed and the starting conformations of the two partners are the bound structures (unbound structures are not available). However, during the simulations only the backbone atoms were restraint to the native structure in order to avoid unfolding of the partners. Allowing for full side chain flexibility, this results in many conformations that do not show perfect interface complementarity. Nevertheless, starting from five different initial random placements (Fig 4) far from the native binding arrangement the RP-REMD-Dock approach sampled in all cases the native binding mode in the reference replica as the dominant configuration (Table 1). This was achieved in some case already after short simulation times (~2 ns) but sometimes required up to 10–20 ns per replica (Fig 4). In the successful cases 20–60% of the sampled states showed an RMSD_lig_ within 4 Å of the native binding site which one can consider as one cluster of solutions. All other sampled placements (40–80%) with larger RMSD_lig_ are forming various other clusters with pairwise RMSD_lig_ > 4 Å. Hence, although not explicitly checked selection of realistic solutions based on the cluster size is likely being a useful approach to screen the results if the native complex structure is not known (as is the case in the present test systems). To test the performance of the REMD method relative to cMD we chose 2 of the starting conformations to perform 10 independent cMD-simulations (with differing initial velocities) of same total length for each structure. This resulted in successful sampling of the near-native binding mode only in a small fraction of cases (Fig 5, Table 2).

**Table 1:** Fraction of near-native protein-protein geometries obtained for the pdb2oo9-homodimer in RP-REMD-DOCK and MD simulations.

	fraction of near native solutions in reference replica	
Start struct. (RMSD_lig_ >25 Å	0–10 ns RMSD_lig_<2 Å (<4 Å)	10–20 ns RMSD_lig_<2 Å (<4 Å)
RP-REMD-DOCK 1 (2)	0.10 (0.43)	0.15 (0.55)
RP-REMD-DOCK 2 (3)	0.06 (0.21)	0.10 (0.28)
RP-REMD-DOCK 3 (5)	0.07 (0.25)	0.20 (0.64)
RP-REMD-DOCK 4 (6)	0.08 (0.23)	0.11(0.34)
RP-REMD-DOCK 5 (7)	0.18 (0.48)	0.24 (0.87)
MD-1 (10 x 20 ns) (3)	0.00 (0.00)	0.00 (0.02)
MD-2 (10 x 20 ns) (6)	0.03 (0.09)	0.04 (0.10)

**Table 2:** Fraction of near-native FKBP51-FK506 geometries obtained in RP-REMD-DOCK and MD simulations.

	fraction of near native solutions in reference replica	
Start struct. (RMSD_lig_ >25 Å	0–10 ns RMSD_lig_<2 Å (<4 Å)	10–20 ns RMSD_lig_<2 Å (<4 Å)
RP-REMD-DOCK 1	0.23 (0.30)	0.56 (0.58)
RP-REMD-DOCK 2	0.00 (0.00)	0.03 (0.33)
RP-REMD-DOCK 3	0.31 (0.34)	0.31 (0.60)
RP-REMD-DOCK 4	0.33 (0.34)	0.34(0.36)
RP-REMD-DOCK 5	0.00 (0.08)	0.39 (0.45)
MD-1 (10 x 20 ns)	0.00 (0.00)	0.00 (0.00)
MD-2 (10 x 20 ns)	0.16 (0.20)	0.20 (0.24)

As a final example using an implicit GB solvent model we investigated the association of a drug-molecule FK506 with the FKBP51 protein. In this case the ligand was fully flexible (no restraints) and only restraints on the backbone of the FKBP protein were included to keep the structure in a folded form. Still the backbone can move by up to 1 Å during the simulations and the side chains are fully flexible. Starting from 5 random starting placements and orientations of the ligand the native binding mode was identified in all RP-REMD-DOCK simulations Table 2). At the final stage the occupancy of near-native placements (within RMSD_lig_< 4 Å) reached 33–60%, indicating that indeed these states form the dominant sampled complexes. However, for this system standard MD simulations could also reach the native binding mode in 3 out of 10 simulations whereas none of the 10 MD simulations starting from the second start site reached the native binding site (Fig 5, Table 2).

**Fig 6:**
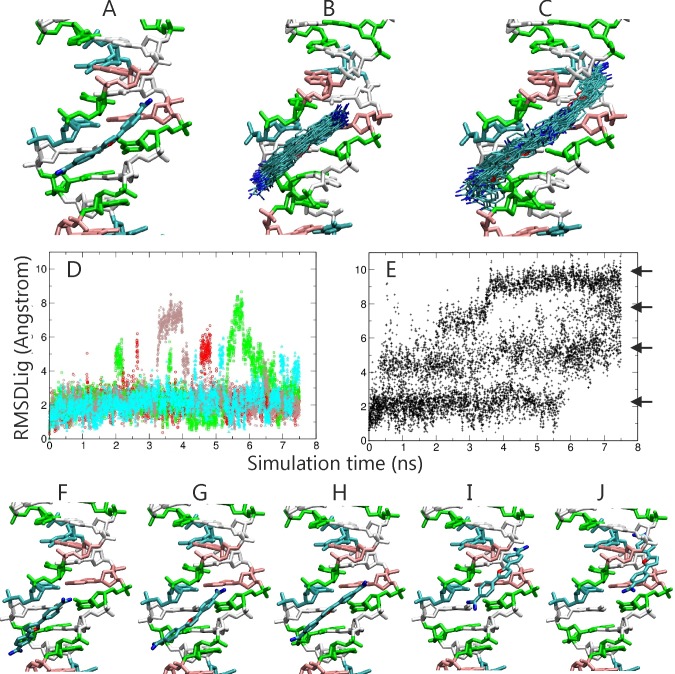
(A) Initial Placement of the DNA minor groove binding ligand BFR at the center of a dsDNA molecule (cyan stick model, the guanine:cytosine base pairs are shown in white:green and the adeneine:thymine in pink cyan color coding, respectively). Hydrogen atoms, explicit waters and ions are omitted for clarity. (B) Superposition of sampled ligand snapshots (75 stick models) obtained from MD simulation and (C) same number of snapshots observed during RP-REMD-Dock in the reference replica. Sampled RMSD_lig_ observed in four example MD simulations (color coded lines in D) and in the REMD reference replica (E). The discrete RMSD_lig_ states are indicated by arrows. (F-J) Snapshots representing clusters of minor groove ligand binding states sampled during RP-REMD-Dock along the DNA minor groove.

In order to investigate the performance of the RP-REMD-DOCK approach in an explicit solvent simulation we performed simulations on a minor groove binding ligand in complex with a dsDNA molecule. The ligand was initially placed in the DNA minor groove approximately at the center of the DNA (Fig 6, see Methods for details). The central part of the DNA contains mainly G:C base pairs whereas the ligand prefers binding to A:T rich segments. Nevertheless, during standard MD simulations the ligand did not move very far from the initial placement due to transient but strong and long-lived hydrogen bonds formed between ligand and DNA in the minor groove. Occasionally, displacements of the ligand along one direction in the minor groove were observed that appeared to occur largely in discrete steps with the ligand moving from one hydrogen bonded state to a neighboring semi-stable state. In case of the RP-REMD-DOCK simulations we observed a much broader sampling of states in the reference replica compared to MD simulations (Fig 6C) and also a sampling of discrete configurations in both directions along the minor groove. The reference replica sampled a state that involved an A:T base pair as the dominant state in the final stage of the RP-REMD-DOCK simulations. However, even for the RP-REMD-DOCK no sampling along the whole DNA minor groove was observed on the time scale of the simulations. Snapshots of the observed sampled states from the RP-REMD-DOCK simulations indicate that transiently stable states are localized at distances along the helical axis of ~3–4 Å, hence, approximately at a distance that corresponds to the base pair distance along the minor groove (Fig 6). This sheds light on the mechanism of sliding motions along the DNA that has been proposed as one possibility to efficiently search for the target binding region. Apparently, such search mechanism involves transient local binding and transition to alternative transiently stable neighboring sites involving significant passage or waiting times before such a movement occurs. The present RP-REMD-DOCK can partially accelerate such transitions because it keeps the ligand slightly apart from the DNA in the higher replicas. Note, that the free diffusion of a minor groove ligand in the aqueous phase is much faster than the movement of the DNA ligands along the minor groove. In principle, the sampling of states in the reference replica allows to extract a probability distribution or free energy of ligand binding along the DNA minor groove. However, our aim is to demonstrate faster search by the BP-REMD-method and much longer simulation times are necessary to achieve a converged distribution.

## Conclusions

In previous studies ultra-long MD simulations have been used to follow the process of ligand-receptor association in explicit solvent and including full conformational flexibility of the binding partners [15,16]. Indeed, MD simulations could be an ideal tool to investigate binding sites and complex formation of two proteins or a drug molecule and a biomolecule. The inclusion of conformational flexibility and the possibility to include solvation effects accurately using either a sophisticated implicit or even explicit solvent model allow the study of a broad range of binding process from weak transient to stable and highly specific binding. However, largely due to transient trapping at locally stable or sticky sites of the protein surface the necessary simulation time to localize all possible binding sites or to reach the globally stable binding region and binding arrangement may still exceed currently available computer resources. Techniques like the reconnaissance metadynamics allow penalizing already visited ligand binding regions to enhance sampling. However, a drawback is that a penalty potential is gradually build up which may slow down the search process. Therefore, the application of MD-approaches in the field of systematic protein-protein or protein-ligand docking is still rare.

It is much more common to identify putative binding sites by means of docking methods. These methods usually include only limited flexibility of the partners or even keep the partners (e.g. in case of protein-protein docking) completely rigid. Moreover, they do not represent water molecules explicitly. We have designed a Hamiltonian replica-exchange MD methodology with a specific biasing potential that aims to prevent “non-specific” sticking of a ligand on the surface of the biomolecule. The biasing potential acts from the beginning of the simulation. Hence, in the higher replicas the ligand can freely move slightly displaced at the surface of the partner and if new promising binding regions are detected the rapid exchange with neighbouring replicas allows a contact evaluation of the binding region in the lower replicas that do not include the biasing potential (or are only weakly biased). The replica exchange approach also guarantees canonical sampling in each replica including the reference replica running under the control of the original force field. A previous H-REMD approach based on softening the interaction between the partners along the replicas was shown to allow efficient refinement of ligand placements starting in the vicinity of the native binding site. The present approach specifically avoids kinetic trapping due to an added repulsive potential and is therefore suitable for a global search of putative binding sites on a receptor surface. The methodology can also be applied in case of ligand binding sites hidden in the interior of a protein. However, there is no extra restraining term that helps to open up such binding sites, our approach only helps to avoid long transient trapping on a pathway to a binding site. In such case a related method that employs an artificial biasing potential between protein atoms and solvent could be useful (35) at least to identify putative buried binding sites that are sampled in replicas with increased solvent-protein interaction.

And there is a second advantage over docking: It is principally possible to use the sampling frequency of the identified binding region to extract the free energy of binding (that includes also the translational, orientational and conformational entropy of the sampled region) instead of only the binding energy. The sampling probability in the reference replica (which effectively includes only a restrain to avoid ligand dissociation beyond a preset cutoff) represents the ligand probability distribution on the receptor surface and hence the relative free energy of binding among the various sampled sites. In principle, with an appropriate reweighting scheme (e.g. like the weighted histogram analysis method) it is also possible to include the sampling distribution in the replicas to improve free energy estimates. However, such effort would require much longer equilibrium sampling beyond the scope of the current study and it will be subject of a future study. For all the systems tested with our RP-REMD-DOCK method we could not only sample a much broader range of binding poses but also identify the native binding mode much faster compared to regular MD simulations. For an artificial peptide-protein and a small protein-protein complex it was possible to identify the native binding mode even starting from starting placements far from the native binding site in reasonable computer time. In the future this may allow REMD based protein-protein complex prediction including full flexible degrees of the binding partners. The possibility of including explicit solvent may allow to identify not only drug-biomolecule interactions at the expected target site but also to study transient semi-stable or alternative specific binding modes including the currently most accurate representation of solvation effects.

## Supporting information

S1 File Zip-archive that contains the necessary code changes in the pmemd program of the Amber package to perform BP-REMD docking simulations and an example application. (ZIP)
